# Knowledge about Vitamin D among Women in the United Arab Emirates

**DOI:** 10.3390/ijerph20021252

**Published:** 2023-01-10

**Authors:** Sharifa AlBlooshi, Fatme Al Anouti, Rafiq Hijazi

**Affiliations:** 1 Department of Health Sciences, College of Natural and Health Sciences, Zayed University, Dubai P.O. Box 19282, United Arab Emirates; 2 Department of Health Sciences, College of Natural and Health Sciences, Zayed University, Abu Dhabi P.O. Box 144534, United Arab Emirates; 3 Department of Mathematics and Statistics, College of Natural and Health Sciences, Zayed University, Abu Dhabi P.O. Box 144534, United Arab Emirates

**Keywords:** Vitamin D, knowledge, women, UAE

## Abstract

Vitamin D deficiency is a significant public health issue as it affects almost 90% of the United Arab Emirates (UAE) population. A bigger portion of this percentage is women due to the physiological changes throughout their life cycle. This study aims to assess the knowledge about vitamin D among women in the UAE. This is a quantitative cross-sectional study. Using snowball sampling, an electronic questionnaire was sent to women aged 18 years and above. Overall, 1537 women completed the questionnaire. The participants had mean scores of 49.4 ± 10.7 and 59.6 ± 14.6 for general and nutritional knowledge, respectively. Factors associated with higher scores included older age, marriage, higher educational level, and having tested their vitamin D levels. Factors associated with lower scores included the history of a chronic illness and being employed. Findings indicate that women in this study showed a low level of vitamin D knowledge specifically regarding the non-skeletal benefits of vitamin D and factors that affect vitamin D synthesis. Therefore, health education and promotion programs must be implemented across the UAE to improve overall awareness about vitamin D.

## 1. Introduction

Vitamin D deficiency is a growing issue around the globe and in the United Arab Emirates (UAE). Studies show that vitamin D deficiency is highest in the Middle East North Africa region [1,2]. In 2017, around 90% of people in the UAE experienced vitamin D deficiency, and this prevalence is continuously increasing [3]. The sun is the primary source of vitamin D but people in the Middle East, including the UAE, typically avoid sun exposure due to the almost year-round high temperatures, as the UAE is a sunny subtropical country [4].

Vitamin D is a fat-soluble vitamin that requires UVB radiation from the sun for cutaneous production. Sunlight is the main source of vitamin D, providing the body with up to 90% of its vitamin D, while other sources such as diet provide only 10% of the body’s needs [5,6,7,8,9]. Having optimal vitamin D levels is highly beneficial for overall wellbeing. Those who are vitamin D deficient tend to experience tiredness, low mood, chronic fatigue, bone pain, back pain, and have an increased risk of generalized anxiety disorder [10,11]. In addition, vitamin D has an essential role in supporting bone health and reducing bone loss and fractures [12]. Moreover, studies found that those with adequate vitamin D levels had fewer airway allergies and infections and improved lung function and glucocorticoid response. On the contrary, coronary heart disease, inflammatory disease, infections, and some cancers were more prevalent among people with vitamin D deficiency. Vitamin D deficient individuals have a 50% higher chance of developing colon, prostate, or breast cancer than people with normal vitamin D levels [13,14]. Additionally, vitamin D supplementation has been associated with better cognitive and nervous system health [14].

Research indicates that vitamin D deficiency occurs more in females than males [4,15,16]. The higher prevalence among women can be attributed to physiological changes such as pregnancy, lactation, menopause, sedentary lifestyle, and low dietary vitamin D consumption [4,17]. Moreover, studies focusing on women as participants had reported that lower education levels, less sun exposure, inadequate vitamin D intake, and low physical activity performance were strongly associated with vitamin D deficiency [8]. Furthermore, women with darker skin complexions were at higher risk since melanin lowers the efficiency of UV-B radiation reaching the skin in the photo-conversion process [18,19]. Other factors include an indoor lifestyle, hot climates, culture, and a lack of social support [20]. Some women also reported concerns over cosmetics, skin health, and skin cancer [21,22]. In the UAE specifically, there are some challenges that prevent women from getting enough vitamin D through sun exposure. For instance, many Emirati women in the UAE wear traditional clothing that requires most of the body to be covered. Therefore, they are less likely to engage in outdoor activities while exposing their skin to sunlight [4,10,14,15,20].

Although vitamin D deficiency is an issue for most people across their lifespan, it is more critical in pregnancy and lactation as it results in unfavorable maternal–fetal outcomes [23]. Insufficient vitamin D levels in women during pregnancy can increase the risk of gestational diabetes, preeclampsia, hypertension, hypocalcemia, and osteoporosis [24,25,26]. Furthermore, a prospective cohort study showed that maternal postpartum depression symptoms were higher in women with low serum 25(OH)D [27]. Additionally, neonates of vitamin D deficient mothers are more prone to experience health complications such as poor growth, low birth weight, and disease [28,29].

Recent guidelines for vitamin D supplementation among the UAE population were published in 2018 suggesting a daily intake for adults of 800–2000 IU all year round, especially in winter. Moreover, pregnant women are advised to consume daily vitamin D supplements of 1500–2000 IU, starting from the first trimester or before trying for a planned pregnancy. These guidelines aim at maintaining optimal serum 25(OH)D level, which is between 30 to 50 ng/mL [30].

In general, previous studies showed that Emirati people were limited in their knowledge about the consequences of vitamin D deficiency on health, vitamin D supplementation, and food fortification. They also had poor attitudes toward sun exposure [4,10,14,15,18,22,31].

This study focuses on addressing the areas of general and nutritional knowledge towards vitamin D among women in the UAE. Concentrating on this group provides new and important insight due to their vulnerability to vitamin D deficiency. In addition to the significance of vitamin D deficiency during pregnancy and lactation to the health of both mother and baby.

## 2. Materials and Methods

### 2.1. Study Design

The study aims to determine the knowledge about vitamin D among women in the UAE. To achieve this, a quantitative cross-sectional study was conducted across the seven Emirates of the country: Abu Dhabi, Dubai, Sharjah, Ajman, Umm Al Quwain, Fujairah, and Ras Al Khaimah.

### 2.2. Sampling

The study included 1537 women from different emirates in the UAE using snowball sampling. Women were recruited to participate in this study by sending them the electronic survey via emails, WhatsApp, Instagram, and Snapchat. The inclusion criteria were English or Arabic- speaking women living in the UAE aged 18 years and older who agreed to participate and completed the survey. In contrast, those under 18 years of age, not living in the UAE, and not willing to participate in the study were excluded. Another exclusion criterion was women who did not complete the survey questions.

### 2.3. Data Collection and Measurements

Data was collected through a structured electronic survey. The questions regarding vitamin D knowledge were adapted from a self-administered questionnaire, tested, and validated earlier then modified to match the study’s purposes [32,33]. The questionnaire included three sections with a total of 21 questions. The first section included 8 questions about socio-demographic information (age, nationality, Emirates and residential area, marital status, menopausal breast feeding or pregnant status, educational level, and employment status).

The second section contained 4 questions about health history, whether they had heard of vitamin D, vitamin D information sources, vitamin D testing, and supplementation. The third section included 9 questions on general knowledge of vitamin D (what are the sources of vitamin D? What is the best source of vitamin D, do you think dietary sources are sufficient to maintain vitamin D levels? Can vitamin D be produced/synthesized in the skin, what factors affect this vitamin D production/synthesis? Are you aware of the updated vitamin D recommendations in the UAE? What are the health benefits of vitamin D? What is the recommended daily amount of vitamin D supplementation for women during pregnancy? Who is most at risk of vitamin D deficiency? And the fourth section has only 1 question on the best food sources of vitamin D [34,35]).

After reviewing relevant articles discussing vitamin D knowledge, it was found that the scoring system for the questions that assess participants’ knowledge of vitamin D created by Boland [36] best suits this study. For such questions, the wrong response was coded as 0, while a correct one was coded as 1.

The response codes for each participant were added up to generate a score for that participant. This score has been ranged from 0 to 9 as there were nine knowledge questions, it was converted to a 100 percent scale for easier reading by dividing it by 9 and then multiplying the result by 100 and the scores were calculated out of 100 [36].

### 2.4. Statistical Analysis

After cleaning and coding the collected data, statistical analysis was performed using IBM SPSS Statistics 28 (IBM, Armonk, NY, USA). Sociodemographic and health-related variables were summarized using frequencies and percentages.

Independent samples *t*-test and One-Way Analysis of Variance (ANOVA) were conducted to compare the general and nutritional knowledge of participating women based on their sociodemographic characteristics. Multiple linear regression models were applied to determine the factors contributing to the general and nutritional knowledge of participants. *p*-values less than 0.05 were considered statistically significant.

### 2.5. Ethical Clearance

This study was approved by the Research Ethics Committee at Zayed University, UAE (ZU21_061_F). All study participants provided informed consent at the beginning of the online questionnaire.

## 3. Results

### 3.1. Sociodemographic Characteristics

The sociodemographic characteristics of the female participants are presented in Table 1. A total of 1537 females were recruited, with most of them (77%) being between 20 to 39 years of age. Moreover, the majority were Emirati (89%) and living in urban settings (88%). The largest percentage of participants were from Abu Dhabi (48%), followed by Dubai (32%), and the Northern Emirates (20%) which include Sharjah, Ajman, Ras al Khaimah, Fujairah, and Umm Al Quwain. In total, 82% of the respondents were married while the rest were divorced, widowed, or separated (18%); (24%) were pregnant; 13% were breastfeeding; and 6.6% were menopausal during the survey period. More than half of the participants were not any of the previous (56%). Regarding education, 58% had earned a university degree, but almost 60% of the participants were unemployed.

Table 2 shows the participants’ health history, vitamin D information source, vitamin D testing, and supplementation data. Around 31% of the subjects had chronic conditions such as migraines, diabetes, and asthma. Interestingly, almost all the participants had previously heard about vitamin D although the sources of information differed. While the main source of information was a health professional (73%), 44% and 42% had their sources from the media and family and friends, respectively. Those who reported previous testing for their vitamin D levels accounted for 77% of the total participants; moreover, those who were taking supplements also accounted for 77%.

### 3.2. Determinants of Vitamin D Knowledge

Table 3 displays the results of comparative tests and multiple regression model for the determinants of general knowledge regarding vitamin D. Overall, the participants had a mean value of 49.4 ± 10.7 for general knowledge indices. There was a significant association between the general knowledge index with age (*p* < 0.001). Older participants (40 and above) had the highest scores. This relation persisted in the multiple regression model analysis (*p* < 0.001) hence confirming that age was a significant determinant for general knowledge about vitamin D among the female participants.

Married females had a higher level of general knowledge compared with those who were not (*p* < 0.001). The multiple regression model analysis also found it a significant predictor of higher general knowledge among the participants (β = 1.73, *p* = 0.018). The level of education was another significant predictor of vitamin D general knowledge (β = 4.62, β = 7.05 and β = 7.32, *p* < 0.001); with those earning higher levels of education having a higher level of knowledge. Being employed predicted lower levels of general knowledge as compared with not being employed (β = −1.5, *p* = 0.009). Similarly, having a chronic condition was a significant predictor of general knowledge (β = −2.35, *p* < 0.001). Thus, females with a medical history were less knowledgeable than healthy females. On the other hand, females who tested their Vitamin D status had higher levels of general knowledge (β = 2.7, *p* < 0.001).

Table 4 illustrates the results of comparative tests and multiple regression models to find the determinants of vitamin D-related nutritional knowledge. The participants had a mean value of 59.6 ± 14.6 for nutrition knowledge indices. As was the case for general knowledge, older age was also associated with better nutritional knowledge. Again, participants older than 40 had the highest mean score (62.2 ± 14.2; *p* = 0.005). Multiple regression also found it a significant determinant for nutrition knowledge regarding vitamin D among the female participants (*p* = 0.008), with only 40+ years female participants possessing a significant difference in nutritional knowledge compared with other age groups (β = 3.7, *p* = 0.036). Abu Dhabi residents had a higher nutrition knowledge index as compared with those residing in the other emirates (60.7 ± 13.7; *p* = 0.021) and this persisted in the multiple regression model (β = 2.32, *p* = 0.021). Marital status was also significantly associated with nutrition knowledge. Married females had a higher nutrition knowledge compared with others (60.1 ± 14.4; *p* = 0.005); however, the multiple regression model was not significant (β = 1.89, *p* = 0.063). Finally, as was seen for general knowledge scores, having a chronic condition was also a significant predictor of lower levels of nutrition knowledge (β = −2.18; *p* = 0.009). In addition, women who had tested their vitamin D levels had higher scores of nutrition knowledge (β = 2.11; *p* = 0.023).

Table A1 and Table A2 in Appendix A list the frequency of each answer for each question regarding the general and nutritional knowledge of vitamin D. In the general questions, when asked about good sources of vitamin D, the common wrong answers included water, air, and exercise. Participants were more knowledgeable about the skeletal benefits of vitamin D, as compared with the non-skeletal benefits. Although 44% stated that they were aware of the updated vitamin D recommendations in the UAE, only 12% knew the correct recommended intake during pregnancy. Incorrect answers remarkably pertained to latitude, season, time of day, sunscreen application, and skin pigmentation in affecting vitamin D synthesis and for being a risk factor. While smoking accounted for a high percentage of correct responses, inaccurate nutrition knowledge was remarkable regarding red meat as only 13% indicated the right answer about red meat as a source of Vitamin D.

## 4. Discussion

Our data revealed that almost 100% of the participants had previous knowledge about vitamin D but the level varied in the context of behavior and attitude. The majority of them at 73% retrieved information about vitamin D from health professionals, 44% from families and friends, and 42% from the media. Similarly, a study by Soliman et al. [32] found that physicians (62%) were the primary source of information for their study sample. This may be due to women seeking antenatal care or visiting a family physician. Furthermore, seeing that our study included 13% lactating mothers and 24% pregnant women, it is a good sign that the majority rely on health professionals instead of other sources to ensure that people receive accurate information on health topics. However, contrasting studies have found that media was the primary source of vitamin D information [22,34].

Those who reported previous testing for their vitamin D levels accounted for 77% of the total participants. This is unlike other studies conducted in the UAE that found vitamin D testing to be uncommon among their participants [15,22,37]. Moreover, 77% of participants were taking supplements. Unlike a study performed in the UAE on female undergraduate students where less than half of the sample were supplementing [37].

On average, women had a low mean general knowledge score of 49.4 ± 10.7 and an average mean score of 59.6 ± 14.6 for nutritional knowledge indices. A slightly lower knowledge score at 39% was reported by Salmanpour et al. [22] who studied the knowledge and practices toward vitamin D among adults in Sharjah.

There was a significant association between both general and nutritional knowledge indices with age where older participants (above 40) scored higher (*p* = 0.001 and *p* = 0.005). This was confirmed by the multiple regression model analysis (*p* = 0.001 and *p* = 0.036). Those with a chronic health condition tended to score lower for both general and nutrition knowledge (β = −3.93, *p* < 0.001 and β = −2.62; *p* = 0.009, respectively). Similarly, a study conducted in east London found better knowledge among the healthy and those older than 65 [35]. Educational level was a significant predictor of vitamin D general knowledge (β = 6.17 and β = 4.95, *p* < 0.001). Participants who earned a university degree had the highest scores. This agrees with research that found that higher levels of education correlate with higher levels of vitamin D knowledge [32].

Our results demonstrated that those who reside in Abu Dhabi had a higher nutrition knowledge index compared with those residing in the other emirates (60.7 ± 13.7; *p* = 0.004) as confirmed by the multiple regression model (*p* = 0.021). Marital status was also important in predicting knowledge. Married females had a higher level of general and nutrition knowledge compared with others (*p* < 0.001 and 0.005, respectively); however only the general knowledge, not the nutrition knowledge, was affected by this variable (β = 2.37, *p* = 0.018). A study in Iraq found higher levels of vitamin D among married women compared with those who were single [38]. This indicates a relationship between being married and improved vitamin D knowledge and practices. 

Being employed was associated with lower scores of general knowledge compared with not being employed (β = −0.261, *p* = 0.009). Additionally, females who tested their vitamin D status had higher levels of both general and nutrition knowledge (β = 2.52 and β = 1.98). Presumably, having tested means that they have been given information from a health professional or they were already aware of the importance of maintaining adequate levels of vitamin D and decided to test. Therefore, they scored higher.

Table A1 and Table A2 in Appendix A show the frequency of each answer for each question regarding the general and nutritional knowledge of vitamin D. Overall, participants commonly answered incorrectly about the good sources of vitamin D, apart from recognizing the sun as a good source. Participants were also more knowledgeable about the skeletal benefits of vitamin D as compared with non-skeletal benefits. Although 44% stated that they were aware of the updated vitamin D recommendations in the UAE, only 12% knew the correct recommended intake during pregnancy. Many answered incorrectly regarding factors that impacted vitamin D synthesis such as latitude, season, time of day, sunscreen application, and skin pigmentation. Inaccurate nutrition knowledge was remarkable regarding red meat as only 13% indicated the right answer about red meat as a source of Vitamin D. In line with our study, several quantitative and qualitative studies at the international, national, and local levels showed a lack of knowledge about vitamin D dietary sources, health benefits, complications related to vitamin D deficiency, and updated vitamin D recommendations [20,32,33,37,39,40,41]. This points to the need for better targeted educational interventions.

Many research articles and clinical practice guidelines have been published in the UAE for the public on the updated vitamin D recommendations. Despite this, our study found that almost half the participants (44%) were not familiar with the updated vitamin D recommendations. Moreover, when asked about the exact recommended daily amount of vitamin D supplementation during pregnancy, only 12% of the participants answered correctly. Likewise, other studies found a lack of awareness about vitamin D recommendations among their study samples [32,36,37]. This suggests that women in the UAE may be less likely to maintain adequate vitamin D status due to a lack of knowledge of the recommendations. Therefore, although there are efforts to educate women on the dangers of vitamin D deficiency [3], educational intervention may need to increase their focus on encouraging corrective actions for vitamin D levels.

### Limitations

There were some limitations to this study. Firstly, this study used snowball sampling to collect the sample, which made the collection quick and easy. However, the use of convenience sampling means that the results of this research cannot be generalized to predict the knowledge and attitudes of the entire UAE female population. Secondly, the use of an online questionnaire as a data measurement tool means that the participants cannot provide in-depth insight into their answers and their reasoning for choosing them. Finally, some aspects were not explored such as the prevalence of vitamin D deficiency among the participants and their attitude toward vitamin D supplementation and fortification.

## 5. Conclusions

Overall, this study highlighted that despite a somewhat average level of knowledge regarding vitamin D among women, practices that negatively impact vitamin D levels persist. Factors associated with higher scores included older age, marriage, higher educational level, and having tested their vitamin D levels. Factors associated with lower scores included the history of a chronic illness and being employed. Findings indicate that women in this study showed a low vitamin D knowledge level specifically regarding the non-skeletal benefits of vitamin D and factors that affect vitamin D synthesis.

Targeting the areas of knowledge that women appear to be lacking has the potential to improve the overall health for mothers and their children. Educational interventions can focus on how to get the recommended amount of vitamin D as per the individual’s needs and the factors affecting vitamin D synthesis.

As this study focused on addressing the areas of knowledge and behavioral patterns toward vitamin D among women in the UAE. We believe the results could incentivize public health professionals and stakeholders to implement interventions focusing on vitamin D education, supplementation, and food fortification strategies as they are considered the most effective ways to prevent vitamin D deficiency [41,42,43,44].

## Figures and Tables

**Table 1:** Sociodemographic characteristics of the respondents (*n* = 1537).

Variable	Categories	Frequency	%
Age	Less than 20 years	105	6.8%
	20–29 years	821	53.5%
	30–39 years	359	23.4%
	40+ years	251	16.3%
Nationality	Emirati	1367	88.9%
	Non-Emirati	170	11.1%
Emirate	Abu Dhabi	739	48.1%
	Dubai	496	32.3%
	Northern Emirates	302	19.6%
Residential Area	Rural	184	12.0%
	Urban	1353	88.0%
Marital status	Married	1262	82.1%
	Divorced/Widowed/Separated	275	17.9%
Currently Breastfeeding, in menopause, or pregnant.	Menopausal	102	6.6%
	Pregnant	366	23.8%
	Breastfeeding	205	13.3%
	Neither	864	56.2%
Educational Level	Less than High School	97	6.3%
	High School Degree	541	35.2%
	Bachelor’s degree	787	51.2%
	Master or PhD Degree	112	7.3%
Employment status	Employed (Full-time, Part-time, Self-employed)	620	40.3%
	Unemployed (Student, Retired, Housewife)	917	59.7%

**Table 2:** Health history, vitamin D information source, and vitamin D testing, and supplementation (*n* = 1537).

Variable	Categories	Frequency	%
Chronic diseases	Migraine headaches	117	7.6%
	Diabetes	109	7.1%
	Asthma and respiratory diseases	87	5.7%
	Hypertension	79	5.1%
	Hypothyroid	76	4.9%
	Arthritis	55	3.6%
	Cancer	43	2.8%
	Parathyroid	36	2.3%
	Kidney diseases	34	2.2%
	Cardiovascular diseases	30	2.0%
Medical history (At least one of the above)	Yes	483	31.4%
	No	1054	68.6%
Previously heard of vitamin D	Yes	1530	99.5%
	No	7	0.5%
Sources of information about vitamin D	Health professionals (doctor, nurse, dietitian, nutritionist)	1127	73.3%
	Media (TV, newspaper, radio, internet, magazine)	681	44.3%
	Family/Friends	638	41.5%
	University	335	21.8%
	Leaflets/Posters	270	17.6%
	Books	212	13.8%
Tested Vitamin D levels	Yes	1183	77.0%
	No	354	23.0%
Taking a vitamin D supplement	Yes	1189	77.4%
	No	348	22.6%

**Table 3:** Determinants of general knowledge.

		Unadjusted Comparison			Regression	
Variables	Frequency	Mean	SD	*p*-Value *	Coefficient	*p*-Value
Age				<0.001		<0.001
Less than 20 years	105	44.3	9.7		Ref	
20–29 years	821	48.4	10.7		2.67	0.017
30–39 years	359	51.2	10.6		5.62	<0.001
40+ years	251	52.2	11.4		7.08	<0.001
Nationality				0.417		
Emirati	1367	49.3	10.8		−1.65	0.054
Non-Emirati	170	50.1	11.8		Ref	
Emirate				0.16		0.107
Abu Dhabi	739	49.9	10.6		0.14	0.843
Dubai	496	48.7	11.0		−1.13	0.146
Northern Emirates	302	49.5	11.5		Ref	
Residential Area				0.077		
Urban	1353	49.6	10.9		1.30	0.119
Rural	184	48.0	11.3		Ref	
Marital status				<0.001		
Married	1262	50.1	10.8		1.73	0.018
Divorced/Widowed/Seperated	275	46.4	11.0		Ref	
Educational Level				<0.001		<0.001
Less than High School	97	43.6	9.6		Ref	
High School Degree	541	48.2	10.3		4.62	<0.001
Bachelor Degree	787	50.7	10.9		7.05	<0.001
Master or PhD Degree	112	50.9	12.5		7.32	<0.001
Employment status				0.194		
Employed (Full-time, Part-time, Self-employed)	620	49.0	10.5		−1.50	0.009
Unemployed (Student, Retired, Housewife)	917	49.7	11.2		Ref	
Medical History				<0.001		
Yes	483	47.4	10.6		−2.35	<0.001
No	1054	50.3	11.0		Ref	
Tested Vitamin D levels				<0.001		
Yes	1183	50.3	10.8		2.70	<0.001
No	354	46.5	10.7		Ref	
Taking a vitamin D supplement				0.872		
Yes	1189	49.4	11.2		−0.83	0.212
No	348	49.5	10.1		Ref	

Dependent Variable: General Knowledge (F = 12.26, *p*-value < 0.001, R-squared = 10.8%). * *p*-values are based on Two-sample *t*-test and One-way ANOVA for dichotomous and multichotomous characteristics, respectively.

**Table 4:** Determinants of nutritional knowledge.

		Unadjusted Comparison			Regression	
	Frequency	Mean	SD	*p*-Value *	Coefficient	*p*-Value
Age				0.005		0.008
Less than 20 years	105	57.5	15.0		Ref	
20–29 years	821	58.9	14.7		0.07	0.963
30–39 years	359	60.1	14.4		0.84	0.621
40+ years	251	62.2	14.2		3.7	0.036
Nationality				0.83		
Emirati	1367	59.6	14.4		−0.87	0.465
Non-Emirati	170	59.9	16.2		Ref	
Emirate				0.021		0.031
Abu Dhabi	739	60.7	13.7		2.32	0.021
Dubai	496	58.9	15.1		0.62	0.568
Northern Emirates	302	58.2	15.8		Ref	
Residential Area				0.924		
Urban	1353	59.6	14.3		−0.45	0.7
Rural	184	59.7	17.0		Ref	
Marital status				0.005		
Married	1262	60.1	14.4		1.89	0.063
Divorced/Widowed/Separated	275	57.2	15.5		Ref	
Educational Level				0.099		0.203
Less than High School	97	56.8	14.1		Ref	
High School Degree	541	59.3	14.7		2.63	0.105
Bachelor Degree	787	59.8	14.1		2.82	0.076
Master or PhD Degree	112	61.7	17.6		4.33	0.036
Employment status				0.294		
Employed (Full-time, Part-time, Self-employed)	620	60.1	14.6		0.87	0.281
Unemployed (Student, Retired, Housewife)	917	59.3	14.6		Ref	
Medical History				0.006		
Yes	483	58.1	14.1		−2.18	0.009
No	1054	60.3	14.8		Ref	
Tested Vitamin D levels				<0.001		
Yes	1183	60.3	14.7		2.11	0.023
No	354	57.1	14.1		Ref	
Taking a vitamin D supplement				0.013		
Yes	1189	60.1	15.0		1.46	0.113
No	348	58.0	13.2		Ref	

Dependent Variable: Nutritional Knowledge (F = 3.49, *p*-value < 0.001, R-squared = 3.33%). * *p*-values are based on Two-sample *t*-test and One-way ANOVA for dichotomous and multichotomous characteristics, respectively.

## Data Availability

Not applicable.

## Appendix A

**Table A1:** General knowledge of vitamin D (*n* = 1537)—Individual Questions.

General Knowledge Questions		Frequency	%
Which of the following are sources of vitamin D?	Food	798	51.9%
	Supplements	864	56.2%
	Sunlight	1263	82.2%
	Water	1435	93.4%
	Air	1498	97.5%
	Exercise	1466	95.4%
What is the best source of vitamin D?	Sunlight	1087	70.7%
Do you think dietary sources are sufficient to maintain vitamin D levels?		592	38.5%
Vitamin D can be produced/synthesized in the skin, what factors affect this vitamin D production/synthesis?	Skin pigmentation	410	26.7%
	Pollution	177	11.5%
	Time of day	371	24.1%
	Latitude	169	11.0%
	Season	266	17.3%
	Smoking	1389	90.4%
	Sunscreen use	311	20.2%
	High-fat diet	162	10.5%
Are you aware of the updated vitamin D recommendations in the UAE?	Yes	678	44.1%
What are the health benefits of vitamin D?	Bone health	962	62.6%
	Prevention of rickets	441	28.7%
	Vision	1240	80.7%
	Hair growth	902	58.7%
	Skin health	895	58.2%
	Prevention of osteoporosis	702	45.7%
What is the recommended daily amount of vitamin D supplementation for women during pregnancy?		183	11.9%
Who are most at risk of vitamin D deficiency?	Individuals not often outdoors (i.e., not out during daylight hours)	853	55.5%
	Institutionalized individuals (i.e., care home)	501	32.6%
	Individuals who cover up majority of their skin when outdoors	450	29.3%
	Individuals with dark skin	148	9.6%
	Individuals who do not eat fish	1215	79.1%

**Table A2:** Nutritional knowledge of vitamin D (*n* = 1537)—Individual Questions.

Nutritional Knowledge Questions		Frequency	%
What are the best food sources of vitamin D?	Oily fish	634	41.2%
	Egg yolks	418	27.2%
	Fortified foods	673	43.8%
	Red meat	195	12.7%
	Dairy products	1290	83.9%
	Fruit	1270	82.6%
	Vegetables	1312	85.4%
	Chicken	1537	100.0%
	Nuts	1360	88.5%
